# Description of a Case of Alveolar Abscess

**Published:** 1842-06

**Authors:** Isaac I. Greenwood


### ARTICLE III.

*Description of a Case of Alveolar Abscess.* By Isaac I. Greenwood, M. D., D. D. S.

Some few years past a foreign gentleman applied to me for professional aid, who had been treated by an eminent surgeon dentist for several years for a diseased dens sapientiæ. On examining the case, the malady was found to be seated in the alveolus of the tooth on the right labial side of the dracranian maxillary at the base of the corenoid process, where it forms a conjunction and continuation of the alveoli. In the first instance it had been formed by an erosive exposure of the medulla of that organ. The patient being of a timid disposition, and the surgeon not determined in extracting the tooth, an abscess had formed, and the pus passing off from the weight of the matter aslantwise, and through the base of the alveolus of the tooth, had perforated the Levator, affecting the rotary muscles opposite the orifice, and through the anterior surface of the skin, immediately under the centre of the belly of the digastricus, where it pierces the meatus auditories externus, forming a considerable orifice; which issue he was in the habit of probing with a silver instrument, about six inches in length, and cleansing with lint, &c. It was found that in making use of this instrument and forcing it in the whole length of the canal, which was straight and considerably indurated, the rigidity being such that the digastricus could not have its full force of expansion, and the masseter muscle of that side at its lower portion was effected as well as the pterygoideus externus in such a manner, that the patient was not enabled to open his mouth more than half an inch.

By further probing the wound the instrument was found to strike upon a hard substance at the base, which by the sound was known to be the fangs of the diseased organ. The alveolus being destroyed on that side of the diacranian opposite, and on the labial section of the surface of the tooth; from the continuous issue of matter, the tooth irritating and acting as an extraneous body, and causing this flow, it was determined at once to perform the operation of extraction. No worse result could take place when the member was removed. The cutting was carefully yet fearlessly made, and the operation performed. The patient immediately feeling relief, the sanguineous discharge which followed was somewhat free, and considered as favourable. Yet still the indurated canal remained, and the rigidity of the parts not remedied. The patient was advised when the wound healed in a measure, to lubricate the parts externally with emollients, such as had been prescribed by his physician and were of a mercurial nature, to cause a pliantness and relaxation of the muscles. The advice was concurred in, and a restitution of the parts was the effect of the application.

